# The Amino Acid Changes T55A, A273P and R277C in the Beta-Lactamase CTX-M-14 Render *E. coli* Resistant to the Antibiotic Nitrofurantoin, a First-Line Treatment of Urinary Tract Infections

**DOI:** 10.3390/microorganisms8121983

**Published:** 2020-12-13

**Authors:** Yasir Edowik, Thomas Caspari, Hugh Merfyn Williams

**Affiliations:** 1 School of Medical Sciences, Bangor University, Brigantia Building, Penrallt Road, Bangor, Gwynedd, Wales LL57 2AS, UK; bsp223@bangor.ac.uk; 2 Faculty of Medicine, Paracelsus Medical University, Strubergasse 21, 5020 Salzburg, Austria

**Keywords:** antibiotic resistance, beta-lactamase, nitrofurantoin, urinary tract infection, CTX-M

## Abstract

The antibiotic nitrofurantoin is a furan flanked by a nitro group and a hydantoin ring. It is used to treat lower urinary tract infections (UTIs) that have a lifetime incidence of 50−60% in adult women. UTIs are typically caused by uropathogenic *Escherichia coli* (UPEC), which are increasingly expressing extended-spectrum beta-lactamases (ESBL), rendering them multi-drug resistant. Nitrofurantoin is a first-line treatment for gram-negative ESBL-positive UTI patients, given that resistance to it is still rare (0% to 4.4%). Multiplex PCR of β-lactamase genes of the *blaCTX-M* groups 1, 2, 9 and 8/25 from ESBL-positive UTI patients treated at three referral hospitals in North Wales (UK) revealed the presence of a novel *CTX-M-14-like* gene harbouring the missense mutations T55A, A273P and R277C. While R277 is close to the active site, T55 and A273 are both located in external loops. Recombinant expression of CTX-M-14 and the mutated CTX-M-14 in the periplasm of *E. coli* revealed a significant increase in the Minimum Inhibitory Concentration (MIC) for nitrofurantoin from ≥6 μg/mL (CTX-M-14) to ≥512 μg/mL (mutated CTX-M-14). Consistent with this finding, the mutated CTX-M protein hydrolysed nitrofurantoin in a cell-free assay. Detection of a novel nitrofurantoin resistance gene indicates an emerging clinical problem in the treatment of gram-negative ESBL-positive UTI patients.

## 1. Introduction

**Figure 1:**
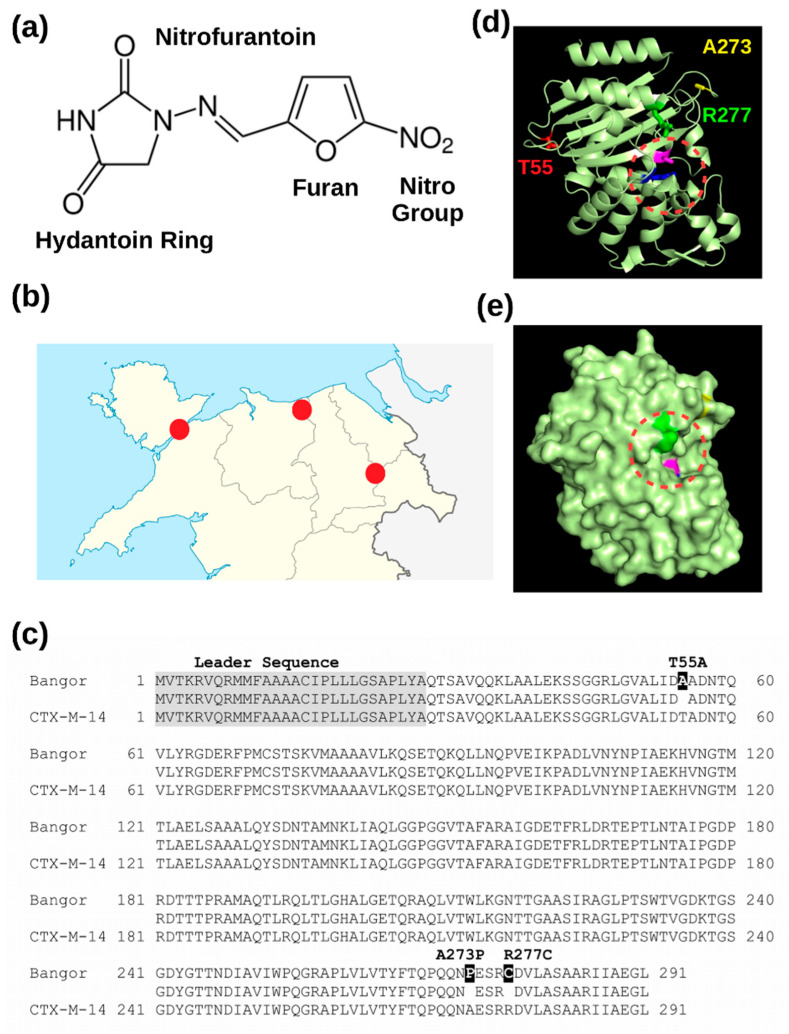
A novel CTX-M beta-lactamase related to *Escherichia coli* CTX-M14: (**a**) structure of the antibiotic nitrofurantoin, (**b**) location of the three hospitals in North Wales, UK, (**c**) alignment of the mutated CTX-M protein and *E. coli* CTX-M14 (AEZ49579.1 (UNIPROT H6UQI0_ECOLX)), (**d**) carton view of *E. coli* CTX-M14 (PDB: 1YLT, active site highlighted by the circle) and (**e**) surface view of the same protein structure.

The antibiotic nitrofurantoin contains a furan ring flanked by a nitro group and a hydantoin ring (Figure 1a). It is used to treat uncomplicated urinary tract infections (UTIs), which are considered the most common outpatient infections around the world with a lifetime incidence of 50−60% in adult women [1]. Most UTIs in non-catheterized older adults are caused by only one bacterial species, typically uropathogenic *Escherichia coli* (UPEC), and more rarely by *Staphylococcus saprophyticus*, or *Enterococcus* [1]. Nitrofurantoin is a first-line antibiotic as only a very low number of UPECs are resistant to it (range: 0–4.4%) [2]. It has been considered a most effective antibiotic against *E. coli* strains, including ESBL producers [3]. Upon its intracellular activation by the nitroreductases NfsA or NsfB, the reduced form of nitrofurantoin reacts with ribosomes, thereby blocking protein synthesis in a not well-understood mechanism [4]. Consistent with the central role of the nitroreductases in the activation of the prodrug form, most nitrofurantoin-resistant *E. coli* carry mutations in the *nfsA* or *nsfB* genes, although overexpression of the OqxAB efflux pump was also detected [5]. We report here the isolation of a novel CTX-M-14-like beta-lactamase that renders *E. coli* cells nitrofurantoin-resistant when recombinantly overexpressed. The novel enzyme differs from CTX-M-14 by only three amino acids (T55A, A273P and R277C). The novel gene was found in 5/45 ESBL-positive *E. coli* isolated from UTI samples from only one referral hospital in North Wales (UK). Interestingly, the gene was absent from a similar number of ESBL-positive UTI samples from two other referral hospitals in this area. The multiple detection of this novel beta-lactamase in a small number of samples indicates an emerging clinical problem as ESBL *E. coli* may acquire this novel beta-lactamase gene by horizontal gene transfer.

## 2. Materials and Methods

### 2.1. Amplification of the CTX-M Genes

UTI isolates were sub-cultured on primary UTI Agar (#2421517) at 37 °C for 18–24 h to identify *Enterococcus spp.*, *Escherichia coli* and coliform bacteria. DNA was isolated, and the CTX-M genes were amplified as described in [6]. Primers: CTX-M group 1: F: 5′-TTAGGAARTGTGCCGCTGYA-3′, R: 5′-CGATATCGTTGGTGGTRCCAT-3; CTX-M group 2: F: 5′-CGTTAACGGCACGATGAC-3, R: 5′-CGATATCGTTGGTGGTRCCAT-3; CTX-M group 9: F: 5′-TCAAGCCTGCCGATCTGGT-3, R: 5′-TGATTCTCGCCGCTGAAG-3; and CTX-M group 8/25: F: 5′AACRCRCAGACGCTCTAC-3, R: 5′-TCGAGCCGGAASGTGTYA3T. DNA fragments were purified and sequenced after cloning into the pJET1.2 plasmid (CloneJET kit/ThermoFischer Scientific/(K1231)).

### 2.2. Construction of CTX-M-14

The CTX-M-14 gene (sequence ID: AEZ49579.1 (UNIPROT H6UQI0_ECOLX)) was obtained by reverting the three amino acids that differ between the mutated CTX-M-14 and *E. coli* CTX-M-14 to the CTX-M-14 sequence using the Q5 Site-Directed Mutagenesis Kit/Thermo Fisher Scientific/(0071605). DNA extracted from isolates 257,294 were cloned in the pASK plasmid and used as a template for the mutagenesis experiment using the following primers:

CTXM-14-A55T-F 5′-ACGCCCAGCCGCCCTCCGTGC-3′
CTXM-14-A55T-R 5′-AGAGATGTGCTGGCCTGGGT-3′
CTXM-14-P273A C277R-F 5′-CCGCAACAGAACGCAGAGAGCCGCAGAGATGTGCTGGC-3′
CTXM-14-P273A C277R-R 5′-CTGGGTAAAATAGGTCACCAG-3′

The reversions were validated by sequencing.

### 2.3. Recombinant Protein Cloning

The CTX-M genes were amplified from the pJET1.2 constructs and inserted into the expression plasmid pASK-IBA2C (IBA 2-1321-000). Cloning of the CTX-M genes into the plasmid pASK-IBA2C results in fusion to a strep tag [7]. All constructs were verified by DNA sequencing. The primers were as follows:

CTX-M-new/14-F: atggcataatggtctcaggccATGGTGACAAAGAGAGTGCAACGG,
CTX-M-new/14-R: atggcataatggtctcagcgctCAGCCCTTCGGCGATGATTC
CTX-M-15-F: atggcataatggtctcaggccATGGTTAAAAAATCACTGCGCCAG
CTX-M-15-R atggcataatggtctcagcgctCAAACCGTCGGTGACGATTTTAG

*E. coli* strain genotype: F-deoR endA1 recA1 relA1 gyrA96 hsdR17(rk−, mk+) supE44 thi-1 phoA Δ(lacZYA argF)U169 Φ80lacZΔM15λ− (Bioline, Bio-85025).

### 2.4. Periplasmatic Protein Expression and Purification

Protein expression and purification were performed as described in [8]. Briefly, one freshly transformed *E. coli* colony was pre-cultured in 5 mL of LB/chloramphenicol (25 µg/mL) at 37 °C (200 rpm, ON). The preculture was then transferred to 20 mL LB/chloramphenicol and incubated at 25 °C until the culture reached an optical density (O.D.) 550 nm of 0.5. Expression was induced by the addition of 10 μL anhydrotetracycline (2 mg/mL in dimethylformamide). After 3 h at 25 °C, cells were harvested (4200 g, 12 min, 4 °C) and the cell pellet was resuspended in 200 mL of cold buffer P (100 mM Tris (pH 8.0), sucrose 500 mM and 1 mM Na2EDTA), incubated on ice for 30 min and then centrifuged for 5 min at 14,000 rpm/Thermo Scientific™ Pico™ 21 Microcentrifuge. The supernatant contained recombinant, periplasmic proteins. The produced recombinant protein was tagged with a short peptide Strep-Tag^®^ II (8 amino acids, WSHPQFEK), which can be genetically fused upstream or downstream from the reading frame of any gene, can be expressed as a fusion peptide and has a high selectivity to Strep-Tactin^®^.

The tagged protein can be purified by the binding affinity between Strep-Tag II and Strep-Tactin using prepacked chromatography columns (iba #18000069), which allows for gravity flow purification of the Strep-Tag fusion proteins. Protein was concentrated on Vivaspin 500 centrifugal filter units (1703013).

### 2.5. MIC Determination

The E-test assay was performed according to the manufacturer’s instructions (BIOMÉRIEUX) by using strips of nitrofurantoin (10047676160), ceftazidime (1004719296), cefoxitin (1004857420), cefotaxime (100495820) and imipenem (100412373)

### 2.6. In Vitro Hydrolysis Assay

The assay was performed as described in [9]. The assay was started by adding 10 μL of purified CTX-M protein (1 μg/μL) to 990 μL of 50 mM phosphate buffer, pH 7.0 containing 5 μM nitrofurantoin at 25 °C in a 1-mL quartz cuvette.

## 3. Results

### 3.1. A Novel Beta-Lactamase Related to CTX-M-14

To characterize the beta-lactamases present in ESBL-positive UTI samples from three referral hospitals in North Wales (UK) (Figure 1b), 100 bacterial strains isolated from UTI samples from each site were sub-cultured; the DNA was extracted; and the beta-lactamase genes of the *blaCTX-M* groups 1, 2, 9 and 8/25 were amplified using multiplex PCR and identified by DNA sequencing. This approach was successful with approximately every second bacterial culture, thus implying that the bacteria in the other UTI samples expressed either no CTX-M gene or a CTX-M gene not detected by the PCR primers. Table 1 summarises the findings from the three hospitals. Further information on the population of UTI patients included in this study is listed in Supplementary Table S1.

CTX-M-15 of the *blaCTX-M* group 1 was the most abundant beta-lactamase amounting to 60%, 64% and 52% of the isolates from the three hospitals, respectively. The dominance of CTX-M-15 is in line with findings from other hospitals in different countries [10,11]. While the diversity of CTX-M genes was lowest in hospital 1, only samples from this hospital were positive for the novel CTX-M gene which was found in five samples. The antibiotic resistance profiles of the strains producing the mutated CTX-M-14 are listed in Table 2.

### 3.2. The Novel Beta-Lactamase Differs in Three Amino Acid Positions from CTX-M-14

The open reading frames of the 5 samples were identical, encoding a novel CTX-M beta-lactamase of 291 amino acids with a N-terminal leader sequence of 28 residues (Figure 1c). A blastp comparison identified CTX-M-14 from Gammaproteobacteria (sequence ID: WP_001617865.1) as the most closely related protein with only three amino acid changes (T55A, A273P and R277C) (Figure 1c). Gammaproteobacteria encompass the group of *Enterobacteriaceae* including *Escherichia coli*. Hence, CTX-M-14 from *E. coli* (sequence ID: AEZ49579.1 (UNIPROT H6UQI0_ECOLX) differs from the new enzyme at the same three 3 positions (T55A, A273P and R277C)). Using the 3D structure of *E. coli* CTX-M-14 as a structural template (PDB-ID: 1YLT [12]), we noticed that arginine R277 is close to the serine and lysine in the active site and may therefore have an impact on substrate recognition. The two other substitutions (T55A and A273R) are located at a distance in a loop each (Figure 1d,e).

### 3.3. Recombinant Expression of the New Beta-Lactamase in the Periplasm of E. coli Renders Cells Nitrofurantoin Resistant

**Figure 2:**
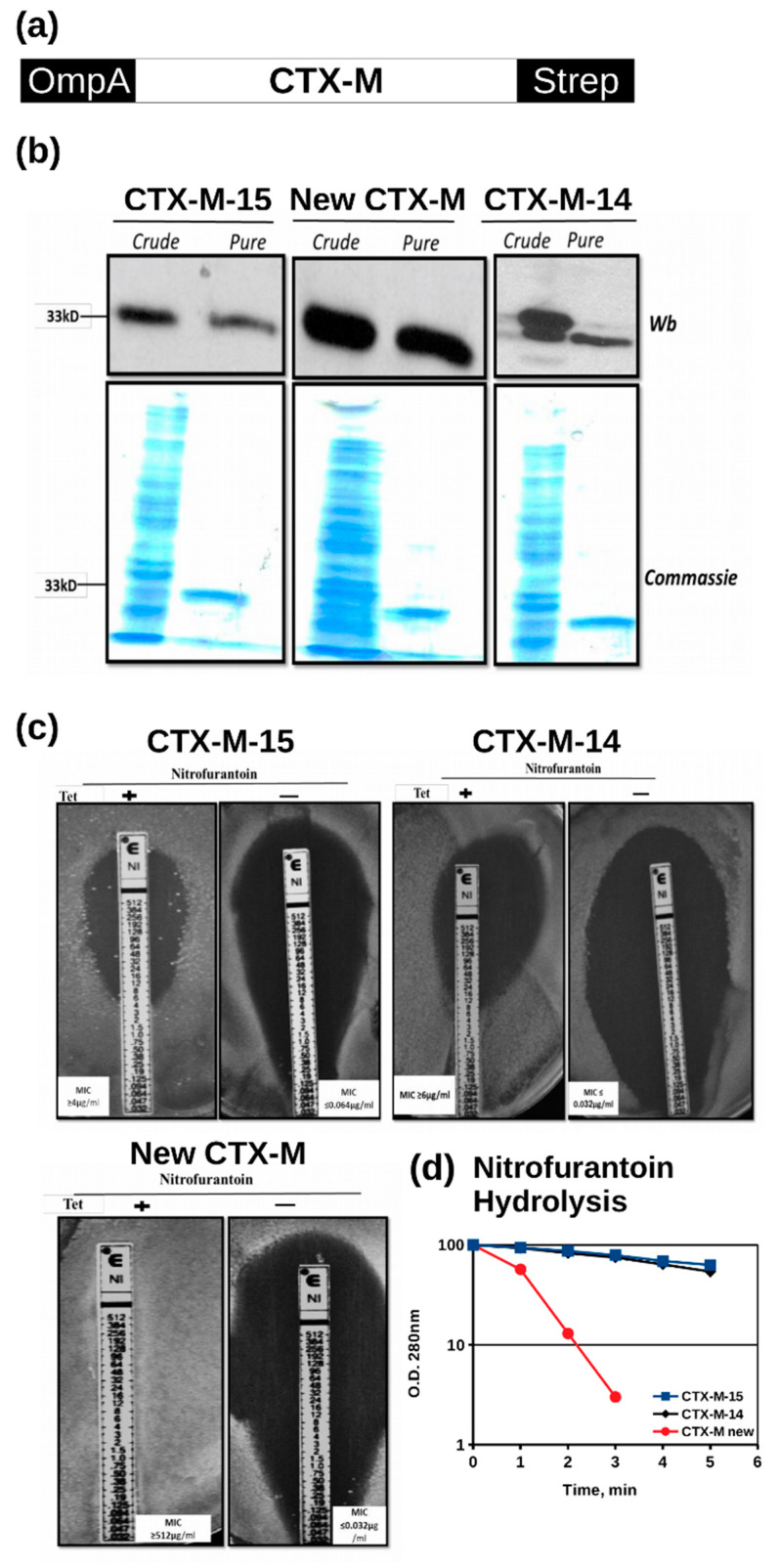
Periplasmatic expression of the new CTX-M-14 renders *E. coli* cells highly nitrofurantoin resistant. (**a**) The new CTX-M gene, *E. coli* CTX-M-15 and *E. coli* CTX-M-14 were cloned between the OmpA leader sequence and the StrepII affinity sequences in the plasmid pASK-IBA-2C; (**b**) induction of the CTX-M proteins by anhydrotetracyclin (Tet +), crude = periplasmic extract, pure = after StrepII affinity purification, Wb = Western Blot with anti-StrepII reagent and Commassie = protein staining; (**c**) Minimal Inhibitory Concentration (MIC) of nitrofurantoin in the E-test, CTX-M-15 4–6 μg/mL), CTX-M-14 (6–8 μg/mL), new CTX-M (≥512 μg/mL), Tet+ = induced, Tet− = not induced; and (**d**) in vitro hydrolysis of 5 μM nitrofurantoin at 25 °C as measured by a decline in nitrofurantoin absorbance at 280 nm by CTX-M-14, CTX-M-15 and the new CTX-M (mean values of three repeats).

To study the activities of the novel beta-lactamase, we cloned the mutated CTX-M-14 gene as well as *Escherichia coli* CTX-M-14 (sequence ID: AEZ49579.1 (UNIPROT H6UQI0_ECOLX)) and *E. coli* CTX-M-15 (sequence ID: ACQ42051.1 (UNIPROT C7S9T0_ECOLX)) into the periplasmatic expression plasmid pASK-IBA2C [7] (Figure 2a). Upon induction of the TetA promoter by anhydrotetracycline, the recombinant beta-lactamases were transported to the periplasmic space of the host *E. coli* strain (Bio-85025), from where they could be readily purified using the C-terminal StrepII affinity tag after osmotic shock (Figure 2b).

Using the E-test assay to establish the minimal inhibitory concentration (MIC) for different tested antibiotics, Imipenem (IP), Cefoxitin (FX), Nitrofurantoin (NI), Cefotazidime (TZ) and Cefotaxime (CT), we noticed a very high resistance to nitrofurantoin of cells expressing of the mutated CTX-M-14 enzyme (Figure 2c). The MIC increased from 0.0032 μg/mL in the absence of the inducer anhydrotetracycline to more than 512 μg/mL in its presence (Figure 2c and Table 3). The second highest increase was found for cefoxitin (0.016 μg/mL to 3.0 μg/mL), whereas the resistance to imipenem, ceftazidime and cefotaxime increased only to a much lesser extent (Table 3). The main difference between CTX-M-14 and the mutated CTX-M-14 was the nitrofurantoin resistance.

To exclude the possibility that the recombinant expression of the mutated CTX-M-14 enzyme renders cells indirectly nitrofurantoin resistant by inactivating the intracellular nitroreductases, which convert the prodrug nitrofurantoin into its active form [4], we measured the ability of the purified CTX-M-15 and CTX-M-14 and the mutated CTX-M-14 to hydrolyse nitrofurantoin in a cell-free assay [9]. Consistent with the MIC test, the mutated CTX-M-14 hydrolysed nitrorurantoin in vitro with a significantly higher rate when compared to CTX-M-14 and CTX-M15 (Figure 2d). Given the low sensitivity of the Coomassie protein staining technique (Figure 2b), we can however not exclude the possibility that a protein co-purifies with the mutated CTX-M14, thus contributing to the in vitro nitrofurantoin hydrolysis.

## 4. Discussion

Taken together, our results support the main conclusion of this work that the mutated CTX-M-14 beta-lactamase, which was isolated from five UTI patients at one referral hospital in North Wales (UK), inactivates the antibiotic nitrofurantoin with high efficiency. The three amino acid changes T55A, A273P and R277C are sufficient to convert CTX-M-14 to an enzyme that hydrolyses nitrofurantoin. This indicates a shift in substrate recognition as nitrofurantoin does not contain a beta-lactam ring (Figure 1a). Horizontal transfer of the new CTX-M gene may therefore become a clinical problem as currently more than 80% of the Enterobacteriaceae isolates from UTI patients are still sensitive to nitrofurantoin [13]. Although the reported frequencies of nitrofurantoin resistance vary in the literature, the overall picture is consistent with a low resistance rate explaining why this antibiotic is still a first-line treatment for UTIs. Given that the mutated CTX-M-14 protein hydrolyses nitrofurantoin also in a cell-free assay (Figure 2d), it is unlikely that the protein renders cells indirectly resistant by blocking the two nitroreductases NfsA and NsfB, which activate the prodrug nitrofurantoin [4]. While the data are in line with our conclusion, there remains however one important point to be discussed. As shown in Table 2, only two out of the five original UTI isolates from which the new CTX-M gene was amplified were nitrofurantoin resistant. One possible explanation for this apparent discrepancy may be the observation that not all beta-lactamase genes are actually expressed in pathogenic bacteria although neither the promotor nor the genes are mutated. For example, 33% of the CTX-M genes including CTX-M-3, CTX-M-15 and CTX-M-24 were silent in clinical isolates of *Klebsiella pneumonia* [14]. The underlying silencing mechanisms are not yet understood but may be linked with the genetic environment or plasmid in which the CTX-M gene resides [11]. As we have amplified the new CTX-M gene by PCR, we have currently no information on its endogenous genetic background in the five original UTI isolates shown in Table 2. We also noticed the absence of CTX-M-14 in our clinical samples although we did find the mutated CTX-M-14 gene five times (Table 1). Given the small number of analysed CTX-M-positive isolates varying between 42 and 45 at the three different hospital sites, it is not possible to conclude that CTX-M-14 is underrepresented.

## Figures and Tables

**Table 1:** Beta-lactamase genes identified at the three hospital sites.

Beta-Lactamase	Hospital 1	Hospital 2	Hospital 3
CTX-M positive samples	45	42	44
Number of different CTX-M genes	12	15	16
CTX-M-15 frequency	60%	64%	52%
	CTX-M genes detected		
CTX-M-1	1	1	1
CTX-M-2	2	1	2
CTX-M-3	0	1	1
CTX-M-9	1	1	4
Mutated CTX-M-14	5	0	0
CTX-M-15	27	27	23
CTX-M-16	0	0	1
CTX-M-27	1	1	2
CTX-M-32	1	1	1
CTX-M-51	0	1	0
CTX-M55	0	1	2
CTX-M-59	0	2	1
CTX-M-65	0	1	0
CTX-M-66	0	1	1
CTX-M-90	1	0	0
CTX-M-108	1	1	1
CTX-M-160	0	0	1
CTX-M-163	1	0	1
CTX-M-172	1	1	0
CTX-M-173	0	0	1
CTX-M-203	0	1	0
CTX-M-225	0	0	1

**Table 2:** Antibiotic resistance spectrum of the original patient samples.

ID	Age	Sex	AMO	CPD	AUG	NIT	TRI	CTX	CAZ	CN	AMI	IMI
226	81	F	R	R	S	S	R	R	R	S	S	S
246	87	F	R	R	S	R	R	R	R	R	S	S
257	91	F	R	R	S	S	R	R	R	R	S	S
283	90	M	R	R	R	R	R	R	R	R	S	S
294	79	M	R	R	S	S	R	R	S	S	S	S

F: female, M: male, R: resistant, S: susceptible, AMO: amoxicillin, CPD: cefpodoxime, Aug: augmentin, Nit: nitrofurantoin, Tri: trimethoprim, CTX: cefotaxime, CAZ: ceftazidime, CN: gentamicin, AMI: amikacin and IMI: imipenem. All bacterial species with the exception of sample 257 were identified as *E. coli*, while sample 257 was identified as coliform.

**Table 3:** Minimum inhibitory concentrations (μg/mL).

*blaCTX-M*	Imi		Cfx		Nit		Caz		Ctx	
Expression	+	−	+	−	+	−	+	−	+	−
CTX-M-15	0.23	0.004	1.8	0,047	3.6	0.064	1.16	0.023	32.0	0.073
CTX-M-14	0.19	0.006	1.0	0.016	6.7	0.032	1.0	0.037	1.5	0.012
CTX-M-new	0.064	0.002	3.0	0.016	512.0	0.037	0.83	0.016	0.75	0.06

Imi = imipenem; Cfx = cefoxitin; Nit = nitrofurantoin; Caz = ceftazidime; Ctx = cefotaxime; +, anhydrotetracycline and −, anhydrotetracyclin, mean values of three repeat experiments.

## Supplementary Materials

The following are available online at https://www.mdpi.com/2076-2607/8/12/1983/s1, Table S1: Characterization of the study isolates (201–300).
