# Supplementary material for: The Amino Acid Changes T55A, A273P and R277C in the Beta-Lactamase CTX-M-14 Render E. coli Resistant to the Antibiotic Nitrofurantoin, a First-Line Treatment of Urinary Tract Infections
Source: Microorganisms. 2020 Dec 13;8(12):1983. doi: 10.3390/microorganisms8121983 (PMC7763680; doi:10.3390/microorganisms8121983)
Supplement: Supplementary file 1 [file microorganisms-08-01983-s001.pdf]

Table S1 Characterisation of the study isolates (201-300)

**Isolate #**	**Age**	**Sex**	**Phenotypic antibiotic**														**ID**	**GP/Hosp**
			AMO	CPD	AUG	NIT	TRI	CTX	CAZ	GEN	AMI	IMI	MER	PTZ	CIP	CPD/CV		
**201**	77	F	R	R	S	S	R	R	R	S	S	S	S	S	S	+	*Colif*	GP
**202**	55	M	R	R	S	S	R	R	S	S	S	S	S	S	R	+	*Colif*	GP
**203**	56	M	S	S	S	S	R	S	S	S	S	S	S	S	S	+	*E. coli*	GP
**204**	88	M	R	R	S	R	R	R	R	S	S	S	S	S	S	+	*E. coli*	Hosp
**205**	72	M	R	R	S	S	S	R	R	R	S	S	S	S	S	+	*E. coli*	Hosp
**206**	71	F	R	R	R	R	R	R	R	S	S	S	S	S	R	+	*E. coli*	GP
**207**	69	F	R	R	S	S	S	R	R	S	S	S	S	S	S	+	*E. coli*	GP
**208**	100	F	R	R	R	S	R	R	R	S	S	S	S	S	R	+	*E. coli*	Hosp
**209**	77	M	R	R	R	R	R	R	R	R	S	S	S	S	R	+	*E. coli*	GP
**210**	75	F	R	R	R	R	R	R	R	R	S	S	S	R	R	+	*E. coli*	GP
**211**	92	F	R	R	R	R	R	R	R	R	S	S	S	S	R	+	*E. coli*	GP
**212**	93	F	R	R	S	R	R	R	R	S	S	S	S	S	R	+	*E. coli*	GP
**213**	90	F	R	R	S	R	R	R	R	R	S	S	S	S	R	+	*E. coli*	GP
**214**	68	M	R	R	R	S	R	R	R	R	S	S	S	R	R	+	*E. coli*	GP
**215**	57	F	R	R	S	S	R	R	R	S	S	S	S	S	R	+	*E. coli*	GP
**216**	59	M	R	R	R	R	R	R	R	S	S	S	S	S	R	+	*E. coli*	GP
**217**	83	F	R	R	R	S	R	R	S	R	S	S	S	R	R	+	*E. coli*	GP
**218**	93	M	R	R	S	R	R	R	R	R	S	S	S	S	S	+	*Prote*	GP
**219**	90	M	R	R	S	S	R	R	R	R	S	S	S	S	R	+	*Colif*	GP
**220**	80	M	R	R	S	R	R	R	R	R	S	S	S	S	S	+	*Colif*	Hosp
**221**	92	F	R	R	S	R	R	R	R	S	S	S	S	S	S	+	*Colif*	GP
**222**	68	M	R	R	R	S	R	R	R	S	S	S	S	S	R	+	*E. coli*	GP
**223**	92	F	R	R	S	S	R	R	R	S	S	S	S	S	R	+	*E. coli*	GP
**224**	85	F	R	R	R	R	R	R	R	R	S	S	S	R	R	+	*E. coli*	GP
**225**	81	M	R	R	S	R	R	R	R	S	S	S	S	S	R	+	*E. coli*	Hosp
**226**	81	F	R	R	S	S	R	R	R	S	S	S	S	S	R	+	*E. coli*	Hosp
**227**	62	F	R	R	S	S	R	R	R	R	S	S	S	S	R	+	*Colif*	Hosp
**228**	52	M	R	R	S	R	R	R	R	S	S	S	S	S	S	+	*Colif*	GP
**229**	70	M	R	R	R	R	R	R	R	S	S	S	S	S	S	+	*Colif*	GP
**230**	62	F	R	R	R	S	R	R	R	R	S	S	S	S	R	+	*Colif*	GP
**231**	97	F	R	R	R	R	R	R	R	R	S	S	S	S	R	+	*E. coli*	Hosp
**232**	40	F	R	R	R	S	R	R	R	R	S	S	S	R	R	+	*E. coli*	GP
**233**	84	M	R	R	S	R	R	R	R	R	S	S	S	S	R	+	*Colif*	Hosp
**234**	96	F	R	R	S	R	R	R	R	R	S	S	S	S	R	+	*E. coli*	GP
**235**	79	F	R	R	S	S	S	R	R	S	S	S	S	S	R	+	*E. coli*	GP
**236**	73	F	R	R	R	R	R	R	R	R	S	S	S	S	R	+	*E. coli*	Hosp
**237**	82	M	R	R	S	S	R	R	S	S	S	S	S	S	R	+	*E. coli*	GP
**238**	86	F	R	R	R	R	R	R	R	R	S	S	S	S	R	+	*E. coli*	GP
**239**	74	F	R	R	R	R	R	R	R	R	S	S	S	R	R	+	*E. coli*	Hosp
**240**	88	F	R	R	R	R	R	R	R	S	S	S	S	S	R	+	*Colif*	GP
**241**	77	F	R	R	R	R	R	R	R	S	S	S	S	S	S	+	*Colif*	Hosp
**242**	66	F	R	R	S	S	R	R	R	S	S	S	S	S	S	+	*E. coli*	GP
**243**	93	F	R	R	R	S	R	R	R	S	S	S	S	S	S	+	*E. coli*	Hosp

			Phenotypic antibiotic															
**Isolate #**	**Age**	**Sex**	AMO	CPD	AUG	NIT	TRI	CTX	CAZ	GEN	AMI	IMI	MER	PTZ	CIP	CPD/CV	**ID**	**GP/Hosp**
**244**	25	F	R	R	R	R	R	R	R	R	S	S	S	S	R	+	*Colif*	Hosp
**245**	74	M	R	R	R	R	R	R	R	R	S	S	S	S	R	+	*E. coli*	GP
**246**	87	F	R	R	S	R	R	R	R	R	S	S	S	S	R	+	*E. coli*	GP
**247**	96	F	R	R	R	R	R	R	R	R	S	S	S	S	R	+	*E. coli*	GP
**248**	25	F	R	R	R	R	R	R	R	R	S	S	S	S	R	+	*Colif*	GP
**249**	91	F	R	R	S	R	R	R	R	R	S	S	S	S	R	+	*Colif*	GP
**250**	92	F	R	R	S	R	R	R	R	R	S	S	S	S	R	+	*Protu*	GP
**251**	96	F	R	R	S	R	R	R	R	S	S	S	S	R	R	+	*E. coli*	GP
**252**	74	M	R	S	S	R	R	R	S	S	S	S	S	S	R	+	*Steno*	Hosp
**253**	94	F	R	R	R	S	S	R	S	R	S	S	S	R	S	+	*E. coli*	GP
**254**	86	F	R	R	S	R	R	R	R	R	S	S	S	R	R	+	*E. coli*	Hosp
**255**	73	M	R	R	R	R	R	R	R	R	S	S	S	R	R	+	*E. coli*	Hosp
**256**	73	M	R	R	R	R	R	R	R	R	S	S	S	R	R	+	*Prote*	Hosp
**257**	91	F	R	R	S	S	R	R	R	R	S	S	S	R	R	+	*Colif*	GP
**258**	71	F	R	R	R	R	R	R	R	R	S	S	S	R	R	+	*E. coli*	GP
**259**	86	F	R	R	S	S	R	R	R	R	S	S	S	R	R	+	*E. coli*	Hosp
**260**	64	F	R	R	R	R	R	R	R	R	S	S	S	R	R	+	*E. coli*	GP
**261**	73	M	R	R	R	R	R	R	R	R	S	S	S	R	R	+	*E. coli*	Hosp
**262**	91	F	R	R	S	R	R	R	R	R	S	S	S	R	R	+	*Colif*	GP
**263**	75	F	R	R	R	R	R	R	R	R	S	S	S	R	R	+	*E. coli*	GP
**264**	88	F	R	R	R	R	R	R	R	R	S	S	S	R	R	+	*E. coli*	Hosp
**265**	73	F	R	R	R	S	R	R	R	R	S	S	S	R	R	+	*E. coli*	GP
**266**	80	F	R	R	R	S	R	R	R	R	S	S	S	R	R	+	*E. coli*	Hosp
**267**	84	F	R	R	R	R	R	R	R	R	S	S	S	R	R	+	*E. coli*	Hosp
**268**	89	F	R	R	R	R	R	R	R	R	S	S	S	R	R	+	*E. coli*	GP
**269**	90	F	R	R	S	S	R	R	R	R	S	S	S	R	R	+	*E. coli*	GP
**270**	73	M	R	R	S	S	R	R	R	R	S	S	S	R	R	+	*E. coli*	Hosp
**271**	22	F	R	R	R	R	S	R	R	R	S	S	S	R	R	+	*E. coli*	GP
**272**	3	F	R	R	S	R	R	R	R	R	S	S	S	R	R	+	*E. coli*	GP
**273**	94	F	R	R	S	S	R	R	R	R	S	S	S	R	R	+	*E. coli*	GP
**274**	20	F	R	R	R	S	R	R	R	R	S	S	S	S	S	+	*E. coli*	GP
**275**	86	M	R	R	R	R	R	R	R	R	S	S	S	R	R	+	*E. coli*	Hosp
**276**	86	F	R	R	R	R	R	R	R	R	S	S	S	R	R	+	*E. coli*	GP
**277**	88	F	R	R	R	R	R	R	R	R	S	S	S	R	R	+	*Colif*	GP
**278**	95	F	R	R	R	R	R	R	R	R	S	S	S	R	R	+	*E. coli*	GP
**279**	89	M	R	R	R	R	R	R	R	R	S	S	S	S	S	+	*Colif*	GP
**280**	80	M	R	R	S	S	R	R	R	R	S	S	S	R	R	+	*E. coli*	GP
**281**	90	M	R	R	S	S	R	R	R	R	S	S	S	R	R	+	*Colif*	Hosp
**282**	80	F	R	R	R	R	R	R	R	R	S	S	S	R	R	+	*Colif*	GP
**283**	90	M	R	R	R	R	R	R	R	R	S	S	S	R	R	+	*E. coli*	Hosp
**284**	49	F	R	R	S	S	R	R	R	S	S	S	S	R	R	+	*E. coli*	GP
**285**	100	F	R	R	R	R	R	R	R	S	S	S	S	R	R	+	*E. coli*	GP
**286**	80	F	R	R	S	S	R	R	R	S	S	S	S	R	R	+	*E. coli*	Hosp
**287**	80	F	R	R	R	R	R	R	R	R	S	S	S	R	R	+	*E. coli*	GP
**288**	20	F	R	R	R	S	R	R	R	S	S	S	S	S	S	+	*E. coli*	GP
**289**	86	F	R	R	S	S	R	R	S	S	S	S	S	R	R	+	*E. coli*	Hosp
**290**	93	F	R	R	R	S	R	R	R	S	S	S	S	R	R	+	*E. coli*	Hosp

Isolate #	Age	Sex	Phenotypic antibiotic														ID	GP/Hosp
			AMO	CPD	AUG	NIT	TRI	CTX	CAZ	GEN	AMI	IMI	MER	PTZ	CIP	CPD/CV		
**291**	50	F	R	R	S	S	R	R	R	S	S	S	S	R	R	+	*E. coli*	GP
**292**	79	M	R	R	R	R	R	R	R	R	S	S	S	R	R	+	*E. coli*	Hosp
**293**	90	F	R	R	R	R	R	R	R	R	S	S	S	R	R	+	*E. coli*	GP
**294**	79	M	R	R	S	S	R	R	S	S	S	S	S	R	R	+	*E. coli*	GP
**295**	76	M	R	R	R	R	R	R	R	S	S	S	S	R	R	+	*E. coli*	GP
**296**	59	F	R	R	R	R	R	R	R	R	S	S	S	R	R	+	*E. coli*	Hosp
**297**	93	F	R	R	S	S	R	R	S	S	S	S	S	R	S	+	*E. coli*	Hosp
**298**	69	M	R	R	R	R	R	R	R	R	S	S	S	R	R	+	*E. coli*	GP
**299**	80	F	R	R	R	R	R	R	R	R	S	S	S	R	R	+	*E. coli*	GP
**300**	79	F	R	R	R	R	R	R	R	S	S	S	S	R	R	+	*E. coli*	Hosp

F: female, M: male, R: resistant, S, Sensitive, Amo: Amoxicillin, CPD: Cefpodoxime, CIP: ciprofloxacin, Aug: Augmentin, Ni: Nitrofurantoin, CTX: cefotaxime, CAZ: Ceftazidime, Gen: Gentamicin, Ami: amikacin, IMI: Imidazole, Mer: Meropenem, Ptz: piperacillin/tazobactam, GP: general practice
